# Qualitative electroencephalogram and its predictors in the diagnosis of stroke

**DOI:** 10.3389/fneur.2023.1118903

**Published:** 2023-06-12

**Authors:** Mohd Syahrul Nizam Ag Lamat, Muhammad Samir Haziq Abd Rahman, Wan Asyraf Wan Zaidi, Wan Nur Nafisah Wan Yahya, Ching Soong Khoo, Rozita Hod, Hui Jan Tan

**Affiliations:** ^1^Department of Medicine, Faculty of Medicine, National University of Malaysia, Kuala Lumpur, Malaysia; ^2^Hospital Canselor Tuanku Muhriz, Jalan Yaacob Latif, Bandar Tun Razak, Kuala Lumpur, Malaysia; ^3^Department of Community Health, Faculty of Medicine, National University of Malaysia, Kuala Lumpur, Malaysia

**Keywords:** qualitative, diagnosis, stroke, predictors, electroencephalography

## Abstract

**Introduction:**

Stroke is a typical medical emergency that carries significant disability and morbidity. The diagnosis of stroke relies predominantly on the use of neuroimaging. Accurate diagnosis is pertinent for management decisions of thrombolysis and/or thrombectomy. Early identification of stroke using electroencephalogram (EEG) in the clinical assessment of stroke has been underutilized. This study was conducted to determine the relevance of EEG and its predictors with the clinical and stroke features.

**Methods:**

A cross-sectional study was carried out where routine EEG assessment was performed in 206 consecutive acute stroke patients without seizures. The demographic data and clinical stroke assessment were collated using the National Institutes of Health Stroke Scale (NIHSS) score with neuroimaging. Associations between EEG abnormalities and clinical features, stroke characteristics, and NIHSS scores were evaluated.

**Results:**

The mean age of the study population was 64.32 ± 12 years old, with 57.28% consisting of men. The median NIHSS score on admission was 6 (IQR 3-13). EEG was abnormal in more than half of the patients (106, 51.5%), which consisted of focal slowing (58, 28.2%) followed by generalized slowing (39, 18.9%) and epileptiform changes (9, 4.4%). NIHSS score was significantly associated with focal slowing (13 vs. 5, *p* < 0.05). Type of stroke and imaging characteristics were significantly associated with EEG abnormalities (*p* < 0.05). For every increment in NIHSS score, there are 1.08 times likely for focal slowing (OR 1.089; 95% CI 1.033, 1.147, *p* = 0.002). Anterior circulation stroke has 3.6 times more likely to have abnormal EEG (OR 3.628; 95% CI 1.615, 8.150, *p* = 0.002) and 4.55 times higher to exhibit focal slowing (OR 4.554; 95% CI 1.922, 10.789, *p* = 0.01).

**Conclusion:**

The type of stroke and imaging characteristics are associated with EEG abnormalities. Predictors of focal EEG slowing are NIHSS score and anterior circulation stroke. The study emphasized that EEG is a simple yet feasible investigational tool, and further plans for advancing stroke evaluation should consider the inclusion of this functional modality.

## 1. Introduction

Stroke remains a significant health burden worldwide. Worldwide, stroke is the second most common cause of death and the third most common cause of disability (1). The worldwide stroke prevalence was 80.1 million in 2016 (1). It carries a tremendous psycho-social burden and a significant impact on health resources. The global stroke burden is expected to rise, especially in developing countries, despite recent advances in stroke prevention, treatment, and rehabilitation (2). In Malaysia, stroke is the third leading cause of mortality after ischemic heart disease and pneumonia (3, 4).

Accurate stroke identification mainly depends on imaging such as computed tomography, perfusion imaging, and magnetic resonance imaging. These investigations provide clinicians with an objective and urgent assessment to select the appropriate patients for specific emergency treatment, particularly intravenous thrombolysis (5) and mechanical thrombectomy (6). Additional angiography (computed tomogram angiography or magnetic resonance angiography) is required to confirm large vessel occlusion for mechanical thrombectomy.

The role of EEG in acute ischemic stroke is somewhat limited as the diagnostic and therapeutic evaluation has been largely dominated by neuroimaging. EEG in stroke may be helpful in post-stroke epilepsy and exclusion of stroke mimics such as seizures and cerebral ischemia (7). Quantitative EEG in stroke showed the power of abnormal, slow activity relative to faster activity and interhemispheric voltage asymmetry (8). Qualitative EEG changes following stroke include focal slowing, generalized slowing, frontal intermittent rhythmic delta activity (FIRDA), and periodic lateralized epileptiform discharges (PLEDs) (9, 10). It has a role in acute and subacute stroke settings as a biomarker for predicting outcomes (11).

It is unclear whether electroencephalographic markers of acute vascular injury severity are independently associated with stroke findings. There needs to be more data on the use and clinical relevance of EEG in acute stroke patients. We embark on this study to evaluate the value of qualitative EEG and its associations with clinical and stroke features.

## 2. Manuscript

### 2.1. Materials and methods

This cross-sectional study was conducted in Hospital Canselor Tuanku Muhriz, the National University of Malaysia, from April 2021 to December 2022 with approval by the local Ethics and Research Board (FF-2021-135). Funding was obtained from the National University of Malaysia. Patients who were admitted with the diagnosis of acute ischemic stroke were recruited using purposive sampling. Patients with debilitating neurological disease, those with severe agitation that prevented a proper EEG recording, stroke mimics (such as patients with underlying seizures, epilepsy, hyperglycemia, metabolic, infection, and venous sinus thrombosis), traumatic brain injury, previous neurosurgery, and tumor were excluded from the study. The patients were diagnosed with acute ischemic stroke following history, clinical examination, and brain imaging. The data on clinical history, type of stroke, demographics, stroke risk factors, and the National Institute of Health Stroke Scale (NIHSS) score were tabulated on admission. The stroke severity was divided according to NIHSS score into mild (1–4), moderate (5–15), moderate to severe (16–20) and severe (21–42). The Oxfordshire Community Stroke Project (OCSP)was used to categorize the type of stroke syndromes which are divided into four subtypes: total anterior circulation infarcts (TACI), partial anterior circulation infarcts (PACI), lacunar infarcts (LACI), and posterior circulation infarcts (POCI) (12).

The EEG was performed as inpatient for all recruited stroke patients and recorded on the Nicolet One Extension (V32 Amplifier) using 24 reusable gold electrodes affixed to the scalp according to the international 10–20 system. The abbreviations on the EEG are as follows: Fp- frontopolar, C- central, F- frontal, T-temporal, P-parietal, O-occipital. Bipolar longitudinal and average referential montages were used for evaluation. The duration of each recording was half an hour. The EEG filter configuration was as follows: 50 Hz filter; low-frequency filter: 0.5 Hz; high-frequency filter: 70 Hz. EEG was evaluated by two trained neurologists blinded to the clinical and radiological findings. Each gave an individual report of the EEG based on the findings. Any discrepancies in the reports were then discussed and the EEG was re-examined and a final joint report was submitted for classification.

Abnormal EEG was defined as a generalized slowing (GS), focal slowing (FS), or presence of epileptiform patterns (spikes, sharp waves, rhythmic and periodic pattern). GS was defined as the dominant rhythm within the theta (4–8 Hz) or delta (< 4 Hz) frequency bands, occurring over all regions of the head. Focal slowing (FS) was defined as slow activity (theta or delta) occurring in a part limited in the area of the head.

Data were explored and analyzed using SPSS software version 21.0. Numerical variables were presented using mean and standard deviation for normally distributed data. The median and interquartile ranges were used for data that were not normally distributed. Categorical variables were presented as frequency and percentage. Distributions of continuous variables were compared using Student’s *t*-tests; Pearson’s chi-square tests or Fisher’s exact tests were used for allocations of categorical variables. A *p*-value less than 0.05 defined statistical significance. Binary and multiple logistic regression was used to determine the risk factors. All odd ratios (ORs) are presented with a 95% confidence interval (CI).

### 2.2. Results

Table 1 shows the demographic data of the study population with a total of 206 participants with a mean age of 64.32 ± 12 years old (range 28–92). Most patients were men (118; 57.28%). The median NIHSS score on admission was 6 (IQR 3-13). According to stroke severity, majority of patients were minor stroke (85, 41.26%), followed by moderate (79, 38.35%) then severe (25, 12.13%), and moderate to severe (17, 8.25%). The most common type of stroke pattern was LACI (103, 50%), then PACI (79, 38.35%), followed by both TACI (12, 5.83%) and POCI (12, 5.83%). By imaging characteristics, most strokes were lacunar stroke (103, 50%), followed by MCA (77; 37.38%,) then PCA (10; 4.85%), infratentorial stroke (9;4.37%), ACA (6; 2.91%) and only one patient with brainstem stroke (1; 0.49%).

**Table 1:** Demographic of the study population.

Baseline characteristics	Results (*n* = 206)
Mean age ± SD (range) (years)	64.32 ± 12 (28–92)
Male	118 (57.28%)
Female	88 (42.72%)
Smoking	59 (28.64%)
**Comorbidities**	
Hypertension	156 (75.73%)
Diabetes mellitus	98 (47.57%)
Dyslipidemia	78 (37.86%)
Atrial fibrillation	23 (11.17%)
Coronary artery disease	19 (9.22%)
NIHSS score	
Median NIHSS score (1. and 3. quartile) (range)	6 (3, 13) (1–35)
**Stroke severity**	
Mild (NIHSS 1–4)	85 (41.26%)
Moderate (NIHSS 5–15)	79 (38.35%)
Moderate to severe (NIHSS 16–20)	17 (8.25%)
Severe (NIHSS 21–42)	25 (12.13%)
**Type of stroke**	
TACI	12 (5.83%)
PACI	79 (38.35%)
POCI	12 (5.83%)
LACI	103 (50%)
**Imaging characteristics**	
MCA	77 (37.38%)
ACA	6 (2.91%)
PCA	10 (4.85%)
Infratentorial	9 (4.37%)
Brainstem	1 (0.49%)
Lacunar	103 (50%)
**EEG**	
Abnormal EEG	106 (51.5%)
Generalized slowing	39 (18.9%)
Focal slowing	58 (28.2%)
Epileptiform	9 (4.4%)

NIHSS, National Institute of Health Stroke Scale; TACI, total anterior circulation infarct; PACI, partial anterior circulation infarct; POCI, posterior circulation infarct; LACI, lacunar cerebral infarct; MCA, middle cerebral artery; ACA, anterior cerebral artery; PCA, posterior cerebral artery.

*Data expressed as frequency and percentage n (%) unless specified.

The EEG was performed within an average of 3.4 days (±3.5 standard deviation). EEG was abnormal in more than half of the patients (106, 51.5%), which constitutes focal slowing (58, 28.2%), followed by generalized slowing (39, 18.9%) and epileptiform (9, 4.4%).

Table 2 shows EEG abnormalities and demographic data of the study population. Patient age significantly affects EEG abnormalities (*p* = 0.002). Similarly, age is associated considerably with generalized slowing (*p* = 0.001). Gender and smoking did not have any association with EEG abnormalities. Among the comorbidities, only hypertension and diabetes mellitus significantly correlate with EEG abnormalities (*p* = 0.012, *p* = 0.035), respectively.

**Table 2:** Demographic data and electroencephalographic abnormalities.

	EEG		*p*-value	Generalized Slowing		*p*-value	Focal slowing		*p*-value	Epileptiform		*p*-value
	Normal	Abnormal		No	Yes		No	Yes		No	Yes	
Number of patients	100 (48.5%)	106 (51.5%)	–	167 (81.1%)	39 (18.9%)	–	148 (71.8%)	58 (28.2%)	–	197 (95.6%)	9 (4.4%)	–
Mean Age ± SD (years)	61.5 ± 12	66.97 ± 12	**0.002**^c^,*	62.96 ± 11	70.13 ± 11	**0.001** ^c,^*	64.36 ± 13	64.21 ± 13	0.938^c^	64.01 ± 13	71.11 ± 10	0.098^c^
Male	60 (29.1%)	58 (28.2%)	0.444^a^	92 (44.7%)	26 (12.6%)	0.188^a^	87 (42.2%)	31 (15%)	0.486^b^	117 (56.8%)	1 (0.5%)	0.005^b^
Female	40 (19.4%)	48 (23.3%)		75 (36.4%)	13 (6.3%)		61 (29.6%)	27 (13.1%)		80 (38.8%)	8 (3.9%)	
**Smoking**												
Yes	35 (17%)	24 (11.7%)	0.050^a^	50 (24.3%)	9 (4.4%)	0.393^a^	45 (21.8%)	14 (6.8%)	0.397^a^	58 (28.2%)	1 (0.5%)	0.451^b^
No	65 (31.6%)	82 (39.8%)		117 (56.8%)	30 (14.6%)		103 (50%)	44 (21.4%)		139 (67.5%)	8 (3.9%)	
**Comorbidities**												
*Hypertension*												
Yes	68 (33%)	88 (42.7%)	**0.012** ^a,^*	123 (59.7%)	33 (16%)	0.15^a^	109 (52.9%)	47 (22.8%)	0.266^a^	148 (71.8%)	8 (3.9%)	0.691^b^
No	32 (15.5%)	18 (8.7%)		44 (21.4%)	6 (2.9%)		39 (18.9%)	11 (5.3%)		49 (23.8%)	1 (0.5%)	
*Diabetes mellitus*												
Yes	40 (19.4%)	58 (28.2%)	**0.035** ^a,^*	74 (35.9%)	24 (11.7%)	0.052^a^	67 (32.5%)	31 (15%)	0.290^a^	95 (46.1%)	3 (1.5%)	0.382^b^
No	60 (29.1%)	48 (23.3%)		93 (45.1%)	15 (7.3%)		81 (39.3%)	27 (13.1%)		102 (49.5%)	6 (2.9%)	
*Dyslipidemia*												
Yes	42 (20.4%)	36 (17.5%)	0.235^a^	64 (31.1%)	14 (16.8%)	0.779^a^	59 (28.6%)	19 (9.2%)	0.344^a^	75 (36.4%)	3 (1.5%)	1.000^b^
No	58 (28.2%)	70 (34%)		103 (50%)	25 (12.1%)		89 (43.2%)	39 (18.9%)		122 (59.2%)	6 (2.9%)	
*Atrial fibrillation*												
Yes	7 (3.4%)	16 (7.8%)	0.065^a^	16 (7.8%)	7 (3.4%)	0.135^a^	15 (7.3%)	8 (3.9%)	0.453^a^	22 (10.7%)	1 (0.5%)	1.000^b^
No	93 (45.1%)	90 (43.7%)		151 (73.3%)	32 (15.5%)		133 (64.6%)	50 (24.3%)		175 (85%)	8 (3.9%)	
*Coronary artery disease*												
Yes	9 (4.4%)	10 (4.9%)	0.914^a^	18 (8.7%)	1 (0.5%)	0.134^b^	11 (5.3%)	8 (3.9%)	0.182^a^	18 (8.7%)	1 (0.5%)	0.589^b^
No	91 (44.2%)	96 (46.6%)		149 (72.3%)	38 (18.4%)		137 (66.5%)	50 (24.3%)		179 (86.9%)	8 (3.9%)	

a Pearson’s chi-square test.

b Fisher’s exact test.

c Independent *t*-test.

d Mann Whitney test.

* Significant, *p*<0.05.

Table 3 shows the stroke characteristics and EEG abnormalities. The median NIHSS score was 10 (4, 19), higher in abnormal EEG compared to 4 (2, 7) in normal EEG (*p* < 0.05). NIHSS score was significantly associated with focal slowing (13 vs. 5, *p* < 0.05). Type of stroke and imaging characteristics were significantly associated with EEG abnormalities (*p* < 0.05). Similarly, focal slowing was also associated with stroke types and imaging characteristics (*p* < 0.05).

**Table 3:** Stroke characteristics and electroencephalographic abnormalities.

	EEG		*p*-value	Generalized Slowing		*p*-value	Focal slowing		*p*-value	Epileptiform		*p*-value
	Normal	Abnormal		No	Yes		No	Yes		No	Yes	
Number of patients	100 (48.5%)	106 (51.5%)	–	167 (81.1%)	39 (18.9%)	–	148 (71.8%)	58 (28.2%)	–	197 (95.6%)	9 (4.4%)	–
**NIHSS**												
Median NIHSS (1. and 3. quartile) (range)	4 (2; 7) (0–25)	10 (4; 19) (0–35)	**<0.05** ^d,^*	6 (3; 13) (0–35)	8 (3; 16) (0–31)	0.324^d^	5 (2; 8.75) (0–31)	13 (5.75; 23) (0–35)	**<0.05** ^d,^*	6 (3; 13) (0–35)	8 (1; 14.5) (0–25)	0.895^d^
**Stroke type**												
TACI	0 (0%)	12 (5.8%)	**<0.05** ^a,^*	11 (5.3%)	1 (0.5%)	0.472^a^	2 (1%)	10 (4.9%)	**<0.05** ^a,^*	11 (5.3%)	1 (0.5%)	0.522^a^
PACI	24 (11.7%)	55 (26.7%)		64 (31.1%)	15 (7.3%)		44 (21.4%)	35 (17%)		74 (35.9%)	5 (2.4%)	
POCI	7 (3.4%)	5 (2.4%)		8 (3.9%)	4 (1.9%)		11 (5.3%)	1 (0.5%)		12 (5.8%)	0 (0%)	
LACI	69 (33.5%)	34 (16.5%)		84 (40.8%)	19 (9.2%)		91 (44.2%)	12 (5.8%)		100 (48.5%)	3 (1.5%)	
**Imaging characteristic**												
MCA	18 (8.7%)	59 (28.6%)	**<0.05** ^a,^*	66 (32%)	11 (5.3%)	0.103^a^	35 (17%)	42 (20.4%)	**<0.05** ^a,^*	71 (34.5%)	6 (2.9%)	0.568^a^
ACA	2 (1%)	4 (1.9%)		4 (1.9%)	2 (1%)		4 (1.9%)	2 (1%)		6 (2.9%)	0 (0%)	
PCA	5 (2.4%)	5 (2.4%)		6 (2.9%)	4 (1.9%)		9 (4.4%)	1 (0.5%)		10 (4.9%)	0 (0%)	
Infratentorial	6 (2.9%)	3 (1.5%)		7 (3.4%)	2 (1%)		8 (3.9%)	1 (0.5%)		9 (4.4%)	0 (0%)	
Brainstem	0 (0%)	1 (0.5%)		0 (0%)	1 (0.5%)		1 (0.5%)	0 (0%)		1 (0.5%)	0 (0%)	
Lacunar	69 (33.5%)	34 (16.5%)		84 (40.8%)	19 (9.2%)		91 (44.2%)	12 (5.8%)		100 (48.5%)	3 (1.5%)	

a Pearson’s chi-square test.

b Fisher’s exact test.

c Independent *t*-test.

d Mann Whitney test.

* Significant, *p*<0.05.

Table 4 shows the multivariate analysis using a logistic regression of EEG findings with patients’ characteristics and stroke features. Older patients have higher odds of getting abnormal EEG (OR = 1.033; 95% CI 1.003, 1.063, *p* = 0.032). Among EEG abnormalities, older patients have higher odds of generalized slowing (OR = 1.067; 95% CI 1.024, 1.111, *p* = 0.002). The male gender is 3.396 times more likely to get generalized slowing on EEG (OR = 3.396; 95% CI 1.350, 8.540, *p* = 0.009).

**Table 4:** The risk factors of electroencephalographic abnormalities in stroke patients.

	Abnormal EEG				Generalized slowing				Focal slowing				Epileptiform			
	Odds ratio	CI 95%		*p*-value	Odds ratio	CI 95%		*p*-value	Odds ratio	CI 95%		*p*-value	Odds ratio	CI 95%		*p*-value
		Lower limit	Upper limit			lower limit	Upper limit			Lower limit	Upper limit			Lower limit	Upper limit	
Age	1.033	1.003	1.063	0.032*	1.067	1.024	1.111	0.002*	0.977	0.944	1.011	0.175	1.034	0.963	1.111	0.357
Gender																
Male	0.836	0.386	1.812	0.651	3.396	1.350	8.540	0.009*	1.285	0.547	3.018	0.565	0.046	0.002	1.217	0.065
Female (reference)																
*Comorbidities*																
Hypertension	1.503	0.645	3.505	0.345	1.128	0.372	3.424	0.831	1.414	0.515	3.880	0.501	3.966	0.339	46.337	0.272
Diabetes mellitus	2.177	1.048	4.524	0.037*	2.242	0.958	5.248	0.063	1.719	0.783	3.773	0.177	0.455	0.093	2.214	0.329
Dyslipidemia	0.59	0.283	1.228	0.158	0.668	0.293	1.521	0.337	0.746	0.325	1.709	0.488	0.583	0.105	3.232	0.537
Atrial fibrillation	1.748	0.545	5.600	0.347	1.769	0.555	5.641	0.335	1.214	0.389	3.788	0.739	0.599	0.059	6.062	0.664
Coronary artery disease	1.046	0.332	3.293	0.938	0.136	0.015	1.231	0.076	3.467	1.068	11.258	0.039*	1.187	0.103	13.642	0.891
Smoking	0.623	0.267	1.454	0.274	0.641	0.237	1.735	0.382	0.778	0.297	2.039	0.610	2.914	0.091	92.789	0.545
NIHSS on presentation	1.090	1.029	1.154	0.003*	1.013	0.959	1.070	0.640	1.089	1.033	1.147	0.002*	0.929	0.823	1.048	0.23
*Stroke type*																
Anterior circulation	3.628	1.615	8.150	0.002*	0.692	0.256	1.871	0.468	4.554	1.922	10.789	0.001*	4.192	0.724	24.282	0.11
Posterior circulation	0.777	0.241	2.500	0.672	2.009	0.582	6.939	0.27	0.389	0.066	2.279	0.295	0	0	0	0.998
Lacunar (reference)																

*Significant, *p* < 0.05.

Patients with diabetes mellitus appeared 2.17 times more likely to have abnormal EEG (OR = 2.177; 95% CI 1.048, 4.524, *p* = 0.037). In looking at the type of EEG abnormalities, patients with coronary artery disease have higher odds of focal slowing (OR = 3.467; 95% CI 1.068, 11.258, *p* = 0.039).

For every increase of 1 point in NIHSS score, there are 1.09 times more likely for abnormal EEG (OR 1.090; 95% CI 1.029, 1.154, *p* = 0.003). Similarly, for every increment in NIHSS score, there are 1.08 times likely focal slowing (OR 1.089; 95% CI 1.033, 1.147, *p* = 0.002). Anterior circulation stroke appeared 3.6 times more likely to have abnormal EEG (OR 3.628; 95% CI 1.615, 8.150, *p* = 0.002). In addition, anterior circulation strokes have 4.55 times higher to have focal slowing (OR 4.554; 95% CI 1.922, 10.789, *p* = 0.01).

### 2.3. Discussion

#### 2.3.1. Incidence of EEG abnormality

In this study, about 51.5% of our patients had an EEG abnormality. In older studies, EEG abnormalities in acute ischemic stroke were reported from 48 to 86% (7, 13). Similarly, a more recent study by Wolf et al. (14) reported 58% of acute ischemic stroke patients had EEG abnormalities. Several studies on post-stroke epilepsy that compared patients with and without seizures quoted 4% (15), 46.2% (16), and 92.4% (17). A significant variation in the findings may be attributed to the type of study, population, and outcome. Several reports (7, 13, 14) on post-stroke epilepsy recruited patients 2–98 months from the onset of stroke (15, 16). EEG abnormalities were also used as predictors of stroke outcomes such as functional status (18–20), post-stroke seizures (16, 17, 21), cognitive decline (22), and mortality (23). Continuous EEG has been performed in 570 consecutive patients in an intensive care unit setting, where 37% were stroke-related, comprised of subarachnoid hemorrhage (19%), intracerebral hemorrhage (7.9%), and ischemic stroke (9.8%) (24). In a subgroup of stroke patients, 26.7% had EEG abnormalities comprising seizures, non-convulsive seizures, and non-convulsive status epilepticus (24).

#### 2.3.2. Types of EEG abnormality

Among the EEG abnormalities in this study, focal slowing was found in 58/106 (54.71%) (Table 1). This figure was lower compared to previous studies, between 75 and 88.6% (13, 14, 18). This could be attributed to the selection of patients as we included different stroke types, from lacunar to territorial stroke. In contrast, other studies recruited mainly territorial stroke consisting of MCA infarct (14, 18). Lacunar infarcts comprised 50% of the stroke subtypes, almost twice higher as a previous study from Malaysia (27.4%) (4). Normal EEG was primarily associated with lacunar infarction in this study.

The second most common abnormality detected was generalized slowing (39/106, 36.79%). Similarly, Holmes et al. (17) reported that 31.6% of acute stroke patients with generalized slowing. On the contrary, a lower percentage of patients with generalized slowing was reported by Wolf et al. (14) (10.2%). The generalized slowing was likely to be found in larger strokes (13, 14). Background activity slowing was significantly associated with poor functional outcomes (18). Thus, the presence of generalized slowing can be determined by the size of the stroke and its prognosis.

Epileptic abnormalities in stroke were infrequently found in a previous study (14). In comparison, we found 4.4% of epileptic potentials in our stroke patients without seizures. Claassen et al. (24) reported 8.9% of acute ischemic stroke with epileptiform discharges. Several studies found epileptiform abnormalities in post-stroke seizures (17, 21, 25). Bentes et al. (18) reported higher epileptiform abnormalities in post-stroke patients with a modified Rankin Scale (mRS) of more than 3. Epileptiform discharges were also observed in critically ill hemorrhagic stroke patients (24).

#### 2.3.3. Associated risk factors for EEG abnormalities

##### 2.3.3.1. Age

EEG abnormalities have been found in the average elderly population comprising generalized slowing, focal slowing, and epileptic discharges (26). The factors associated with EEG abnormalities in a cohort were known epilepsy/seizure and structural brain lesion (27). Our study has a similar observation that age was a risk factor for the occurrence of EEG abnormalities post-stroke. Wolf et al. (14) identified older stroke patients with a significantly higher proportion of abnormal EEG. EEG abnormalities and age were independent predictors of functional outcome (18) and dementia (22). This study determined that age was also a risk factor for generalized slowing. Contrary to our finding, generalized slowing was not associated with age in a study by Wolf et al. (14). The presence of alpha/theta coma and generalized suppression were associated with worse clinical outcomes of stroke (28).

##### 2.3.3.2. Male gender

In a systemic review, the incidence of stroke in males was 33% higher, and stroke prevalence was 41% higher than the females (29). Similar changes were also observed in a previous study in the Malaysian stroke population that showed 55% were males (4), comparable to our study (57%). Despite a higher proportion of male compared to female patients, previous studies in stroke did not reveal any significant gender differences in EEG changes (14, 18). However, our study showed males had a 3.39 higher odds ratio to have generalized slowing. This may be attributed to our larger sample population of 206 patients to achieve a significant result. Furthermore, 75% of the patients had hypertension. Appelros et al. (29) highlighted the incidence rates of brain infarction were higher among men, although stroke severity was higher in women. An experimental study on male hypertensive rats showed profound brain atrophy at 12 weeks post-stroke, possibly attributed to increased apoptosis (30). Brain atrophy has been linked with EEG changes in dementia (31). Further evaluation of the underlying mechanisms may yield gender disparities in EEG alterations.

##### 2.3.3.3. Diabetes mellitus

Diabetes mellitus contributed to 47.57% of our study population, similar to the incidence of diabetes in the Malaysian stroke population in an earlier report (45%) (4). Both traditional risk factors, such as hypertension and diabetes mellitus, have been identified in Asian and Western countries (32). The rationale of studying the association of EEG findings in stroke patients with diabetes mellitus is largely attributed to the large population of diabetes in our cohort. Stroke patients with diabetes mellitus were found to have a significant association with EEG abnormalities. In the logistic regression analysis, stroke patients with diabetes mellitus are 2.17 times more likely to have abnormal EEG. Diabetes mellitus contributes to stroke as it increases the risk of cerebral ischemia and atherosclerotic changes in the cerebral circulation. There is an increased risk of different subtypes of ischemic stroke including lacunar, large artery occlusion and thromboembolic strokes (33). Mechanisms for these causative factors were identified as reduced cortical functional connection and hyperglycemia-induced changes (34). Brain dysfunction in diabetes mellitus has been attributed to glucose homeostasis impairment which is responsible for elevated oxidative damage (35). However, the underlying pathology of EEG abnormalities in diabetic stroke patients needs to be further ascertained.

#### 2.3.4. NIHSS and EEG abnormalities

The stroke severity in terms of a higher NIHSS score is more likely to have abnormal EEG. In most cases, the NIHSS score corresponds to stroke size similar to our study. This was in line with previous studies of EEG changes found in larger ischemic lesions (7, 36). EEG changes correlate with the severity of initial clinical findings (14, 36) and also clinical deterioration of stroke (14). NIHSS score and abnormal EEG have also been independently associated with functional outcomes (18). Prediction of acute stroke evolution can be monitored using sensitive quantitative EEG data (11). The functional outcome of a stroke at 6 months can be indicated by a higher NIHSS score and quantitative EEG analysis such as derived Brains Symmetry Index (dBSI) and (delta + theta)/(alpha + beta) ratio (DTABR) (23).

Our study on qualitative EEG demonstrated that an increment in NIHSS score had increased the risk of developing focal slowing. Such finding was also reported in acute stroke settings where a higher NIHSS score was associated with focal slowing (14). However, in the same study, stroke deterioration was associated with abnormal EEG and generalized slowing (14). To date, only a few studies have observed an association between NIHSS score and focal slowing. Furthermore, quantitative EEG and stroke studies showed a significant correlation between NIHSS score at 30 days with acute delta change index (aDCI) (11) and delta alpha ratio (DAR) (37). The neurophysiological alterations post mono hemispheric stroke has been studied using spectral exponent (SE). Spectral exponent which is part of quantitative EEG, reflects EEG slowing and quantifies the power-law decay of the EEG Power Spectral Density (PSD). The study showed that stroke patients had significantly more negative SE values in over the affected hemisphere than healthy control and SE renormalization significantly correlated with NIHSS improvement (38).

#### 2.3.5. Anterior circulation and EEG abnormalities

Focal abnormalities on EEG following cortical infarct depend on the location of the vascular territory. Anterior circulation stroke in our patients appeared to have 3.63 higher odds of having an abnormal EEG. Comparing the other vascular regions, anterior circulation stroke has 4.55 times to develop focal slowing. A previous study showed that 44.9% of patients with anterior circulation stroke had abnormal EEG (14). Lateralized EEG abnormalities were observed in 80% of cortical middle cerebral artery territory infarct and 86% of cortical watershed infarct (13). Focal slowing has a role in determining the functional outcome, as indicated by 92.9% of MCA stroke patients with mRS of more than three compared to 80.8% of patients with mRS of less than 3 (*p* = 0.025) (18). Quantitative EEG has been used in studies to determine larger infarcts and diagnosis of stroke and large vessel occlusion (39). Larger infarct size was associated with higher aDCI (11) and lower beta power (40). The presence of delta band power and alpha/delta frequency band ratio significantly distinguished patients with large acute ischemic stroke from all other suspected stroke cases (41). Although neuroimaging techniques have surpassed EEG for the diagnosis of stroke, there is still a role for EEG in resource limited settings where brain imaging is not readily available. Detection of focal slowing on the EEG may use to predict the location of the vascular territory and hence, the management of stroke may be tailored accordingly.

#### 2.3.6. Type of EEG monitoring

This study used the standard EEG montage of 24 electrodes and the recordings take approximately 15–20 min to complete the data acquisition. The EEG were performed at the average of 3.4 days on the stroke patients. However, in acute stroke settings which are time-dependant, this procedure may not be feasible. A more viable approach can be carried out using a wearable portable EEG device (42). One study found that the Muse headband that uses seven electrodes reported increased brain asymmetry in stroke patients compared to the healthy controls (43). Additionally, moderate and severe strokes showed increased delta to alpha ratio (DAR) and increased (delta + theta)/(alpha + beta) ratio, (DTABR) (44). The application of quantitative EEG using pairwise-derived Brain Symmetry Index (pdBSI) and measures of slowing can be implemented in a prehospital setting with automated interpretation.

### 2.4. Conclusion

The stroke assessment largely depends on clinical examination and neuroimaging techniques to identify the ischemic core and salvageable penumbra. The role of EEG in providing functional monitoring for stroke can be considered. EEG can be viewed as a complementary investigative tool as part of the workup for diagnosing stroke. These EEG predictors allow the clinician to determine the vascular territory and the severity of the stroke especially in the resource-limited settings.

### 2.5. Limitation of study

The following reasons limited this study: The sample population was only recruited from a single center, and the timing of the EEG was variable and not standardized among the patients. Continuous EEG was not performed due to limitations in time and laboratory personnel. There was a limited follow-up for these patients for further complications such as hemorrhagic transformation or seizures. The standard EEG uses 24 electrodes that may require additional time for the procedure and thus, may not be practical in acute stroke patients.

## Data availability statement

The original contributions presented in the study are included in the article, further inquiries can be directed to the corresponding author.

## Ethics statement

The studies involving human participants were reviewed and approved by Faculty of Medicine, National University of Malaysia. The patients/participants provided their written informed consent to participate in this study.

## Author contributions

MoA and HT: conceptualization, methodology, investigation, formal analysis, and writing-original draft. MoA: investigation, formal analysis, and writing-original draft. WW: methodology, investigation, formal analysis, and writing-review and editing. WY: methodology, investigation, and writing-review and editing. CK: data acquisition, formal analysis, and writing-review and editing. MuA: data acquisition and writing-review and editing. RH and HT: methodology and writing-review and editing. All authors contributed to the article and approved the submitted version.

## Funding

This study was funded by the Faculty of Medicine, The National University of Malaysia research grant (FF-2021-135).

## Conflict of interest

The authors declare that the research was conducted in the absence of any commercial or financial relationships that could be construed as a potential conflict of interest.

## Publisher’s note

All claims expressed in this article are solely those of the authors and do not necessarily represent those of their affiliated organizations, or those of the publisher, the editors and the reviewers. Any product that may be evaluated in this article, or claim that may be made by its manufacturer, is not guaranteed or endorsed by the publisher.

## Glossary

NIHSS: National Institutes of Health Stroke Scale
EEG: Electroencephalography
FIRDA: Frontal intermittent rhythmic delta activity
PLEDs: Periodic lateralized epileptiform discharges
OCSP: Oxfordshire Community Stroke Project
TACI: Total anterior circulation infarct
PACI: Partial anterior circulation infarct
LACI: Lacunar infarct
POCI: Posterior circulation infarct
GS: Generalized slowing
FS: Focal slowing
ACA: Anterior cerebral artery
MCA: Middle cerebral artery
PCA: Posterior cerebral artery
OR: Odds ratio
CI: Confidence interval
aDCI: Acute delta change index
DAR: Delta alpha ratio
mRS: Modified Rankin Scale
dBSI: Derived Brains Symmetry Index
DTABR: (Delta + theta)/(alpha + beta) ratio
